# Combined analysis of the metabolome and transcriptome provides insight into seed oil accumulation in soybean

**DOI:** 10.1186/s13068-023-02321-3

**Published:** 2023-04-25

**Authors:** Xunchao Zhao, Jie Wang, Ning Xia, Yuanyuan Liu, Yuewen Qu, Meng Ming, Yuhang Zhan, Yingpeng Han, Xue Zhao, Yongguang Li

**Affiliations:** Key Laboratory of Soybean Biology in Chinese Ministry of Education (Key Laboratory of Soybean Biology and Breeding/Genetics of Chinese Agriculture Ministry), Northeast Agricultural University, Harbin, 150030 China

**Keywords:** Oil content, Transcriptome, Metabolome, Soybean

## Abstract

**Background:**

Soybean (*Glycine max* (L.) Merr) is an important source of human food, animal feed, and bio-energy. Although the genetic network of lipid metabolism is clear in *Arabidopsis*, the understanding of lipid metabolism in soybean is limited.

**Results:**

In this study, 30 soybean varieties were subjected to transcriptome and metabolome analysis. In total, 98 lipid-related metabolites were identified, including glycerophospholipid, alpha-linolenic acid, linoleic acid, glycolysis, pyruvate, and the sphingolipid pathway. Of these, glycerophospholipid pathway metabolites accounted for the majority of total lipids. Combining the transcriptomic and metabolomic analyses, we found that 33 lipid-related metabolites and 83 lipid-related genes, 14 lipid-related metabolites and 17 lipid-related genes, and 12 lipid-related metabolites and 25 lipid-related genes were significantly correlated in FHO (five high-oil varieties) vs. FLO (five low-oil varieties), THO (10 high-oil varieties) vs. TLO (10 low-oil varieties), and HO (15 high-oil varieties) vs. LO (15 low-oil varieties), respectively.

**Conclusions:**

The *GmGAPDH* and *GmGPAT* genes were significantly correlated with lipid metabolism genes, and the result revealed the regulatory relationship between glycolysis and oil synthesis. These results improve our understanding of the regulatory mechanism of soybean seed oil improvement.

**Supplementary Information:**

The online version contains supplementary material available at 10.1186/s13068-023-02321-3.

## Background

Soybean (*Glycine max* (Linn.) Merr) is an important crop that produces high-quality protein and vegetable oil [1]. To ensure a global supply of soybean products, the development of high-yield and high-oil cultivars has become the primary breeding target in soybean breeding programs [2]. Soybean is rich in various primary and secondary metabolites, such as flavonoids, lipids, and sugar metabolites [3, 4]. Lipid metabolites play an important role in seed metabolism and are the main carrier for the production of soybean oil [5]. In recent years, the functional genes related to plant primary and secondary metabolism have been determined by metabolomic and multi-omics analyses [6, 7]. A systematic analysis of the lipid species and content and an understanding of the associated molecular mechanisms in soybean are important for lipid metabolism research in soybean.

Non-targeted metabolomics can be used to detect a wide array of metabolites and has been applied to plant, microbiology, and animal research [8]. In-depth research into metabolites can further our understanding of key regulatory substances and can determine cellular processes with metabolic balance [9, 10]. Non-targeted metabolomics approaches have been applied to multiple species. Qin et al. applied an ultra-high-performance liquid chromatography coupled with Linear Trap Quadrupole and OrbiTrap MS (UHPLC-LTQ-OrbiTrap-MS) metabolomics approach to analyze the characteristic metabolites between tea varieties, identifying 90 differential metabolites [11]. Previous research detected 90 flavonoid-related metabolites in quinoa seeds, including 18 metabolites that were important contributors to flavonoid biosynthesis [12].

Plant seed oils are generated in the endoplasmic reticulum (ER) and are stored as triacylglycerols (TAGs) [13, 14]. The precursors of TAGs are mainly derived from glycolysis, and glycolysis is catalyzed via enzymes to generate acyl-CoAs [15, 16]. The acyl-CoAs are assembled in glycerol diaphysis to form TAGs via the Kennedy pathway [17]. Glycerol-3-phosphate (G3P) also acts as a precursor for TAG assembly at the ER. The G3P is gradually acylated by a series of enzymes to convert TAGs, involving glycerol phosphate acyltransferase (GPAT), lysophosphatidic acid acyltransferase (LPAAT), diacylglycerol acyltransferase (DGAT), and phospholipid acyltransferase (PDAT) [15–18].

In recent years, researchers have made progress in the study of lipid metabolism. In *Arabidopsis*, the over-expression of *AtDGAT* increased the seed oil content and seed weight [19]. The over-expression of flax *LuDGAT1*, *LuPDAT1,* and *LuPDAT2* in *Arabidopsis* significantly increased the oil content of seeds [20]. In *Arabidopsis*, *AtPDAT1* silenced by RNA-interference (RNAi) in a *dgat1-1* condition or *AtDGAT1* silenced by RNAi in a *pdat1-1* condition resulted in a 70–80% decrease in seed oil content [21]. Studies have shown that *MYB89* inhibits the accumulation of oil content in seeds, and the *MYB89* knockdown were found to increase oil content significantly [22]. The over-expression of *BnWRI1* in *Arabidopsis* increased the oil content of the seed by approximately 10–40% [23]. Previous research found that the mutation of *WRKY6* led to a significant increase in seed size and a higher percentage of oil bodies in the mature seeds of *Arabidopsis* [24].

There is few comprehensive combined non-targeted metabolomics and transcriptomics analysis of the lipids in soybean seeds. In this study, a combined metabolome and transcriptome method was used to explore the regulatory mechanism of lipid metabolism in soybean seeds. Based on the combined analysis, we identified lipid-related metabolites associated with oil synthesis and further revealed the regulatory relationship between glycolysis and oil synthesis. The results are important for understanding oil accumulation in soybean.

## Results

### Soybean oil contents and metabolic profiling analysis

To identify a comprehensive lipid regulatory network at the seed development stage, we used non-targeted metabolic profiling analysis. Thirty soybean varieties were included in this experiment, comprising 15 high-oil (HO) and 15 low-oil (LO) soybean varieties (Additional file 1: Table S1, Figure S1). A total of 5970 metabolites were identified in at least one soybean sample, including organic acids, amino acids, phenylpropanoids, secondary metabolites, lipids, and flavonoids.

**Fig. 1:**
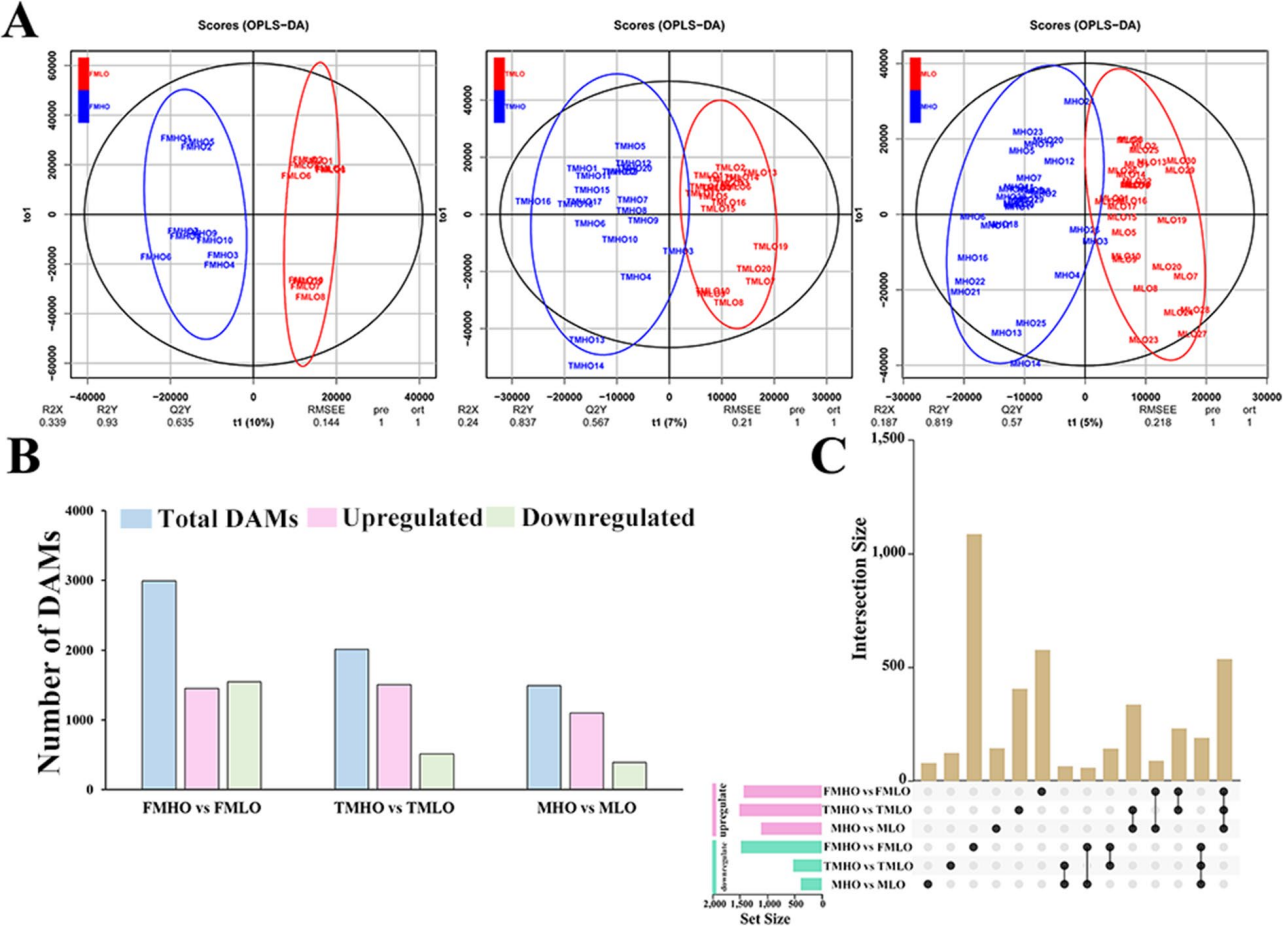
Multivariate statistical analysis of the metabolome data in the soybean samples. **A**. OPLS-DA model analysis. **B**. Number of differential metabolites in different comparison groups. Pink and green columns represent the numbers of genes with upregulated and downregulated expression, respectively. **C**. Upsetplot diagram showing the overlapping DAMs in the three comparison groups

To identify the differences in metabolites between different varieties, three comparison groups were defined, namely a comparison of the five high-oil (FMHO) and five low-oil (FMLO) varieties (FMHO vs. FMLO); a comparison of 10 high-oil (TMHO) and 10 low-oil (TMLO) varieties (TMHO vs. TMLO); and a comparison of 15 high-oil (MHO) and 15 low-oil (MLO) varieties (MHO vs. MLO). As shown in Fig. 1, the OPLS-DA found that the model performed relatively well and could accurately describe the samples (Fig. 1A). Based on the OPLS-DA model, a total of 1448 differentially abundant metabolites (DAMs) were upregulated, and 1545 DAMs were downregulated in FMHO vs. FMLO. These metabolites included flavonoids, amino acids, lipids, and unknown metabolites. Furthermore, a total of 2015 DAMs and 1491 DAMs were identified in TMHO vs. TMLO and MHO vs. MLO, respectively (Fig. 1B). As shown in Fig. 1C, there were 535 common upregulated DAMs and 188 common downregulated DAMs identified. Metabolite annotation showed that a total of 98 metabolites were identified in the metabolome to participate in lipid synthesis.

### Transcriptomic analysis of HO and LO soybean seeds

To understand the transcriptional regulation of lipid metabolism in HO and LO soybean seeds, RNA-seq analysis was performed. Three comparison groups were identified, namely a comparison group of five HO and five LO varieties (FHO vs. FLO); a comparison of 10 HO and 10 LO varieties (THO vs. TLO); and a comparison of 15 HO and 15 LO varieties (HO vs. LO).

**Fig. 2:**
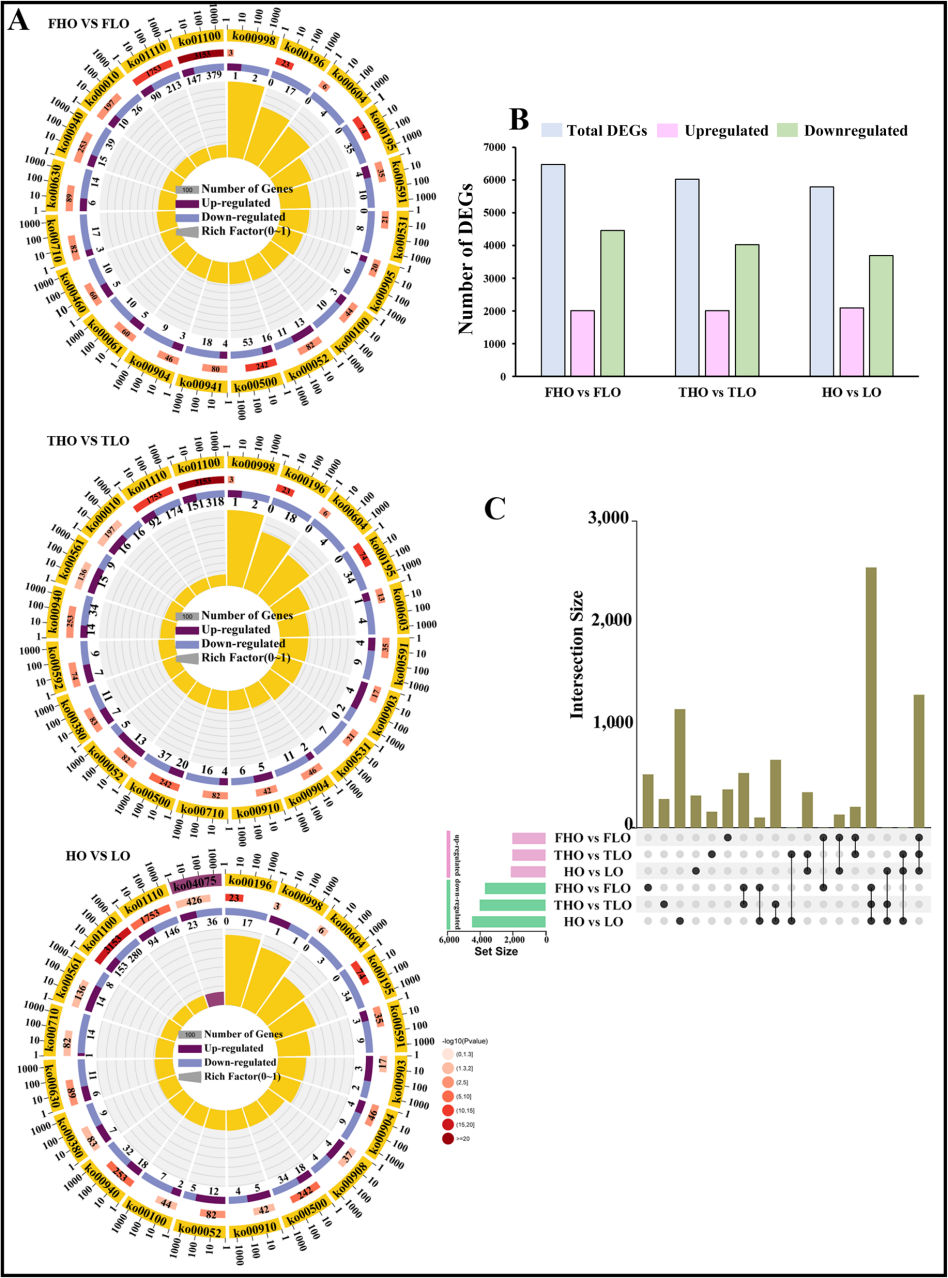
Multivariate statistical analysis of the transcriptome data in the soybean samples. **A** KEGG enrichment of DEGs. **B** Number of differential genes in different comparison groups. Pink and green columns represent the numbers of genes with upregulated and downregulated expression, respectively. **C** Upsetplot diagram showing the overlapping DEGs in the three comparison groups

According to differential expression analysis, there were 6470, 6025, and 5783 DEGs in FHO vs. FLO, THO vs. TLO, and HO vs. LO, respectively. The numbers of upregulated DEGs were higher than the numbers of downregulated DEGs in the comparison groups, except for FHO vs. FLO (Fig. 2A, B). As shown in Fig. 2C, a total of 1299 common DEGs were found with upregulated expression, while 2542 common DEGs were identified with downregulated expression.

### Differential lipid-related DEGs in HO and LO soybean seeds

**Fig. 3:**
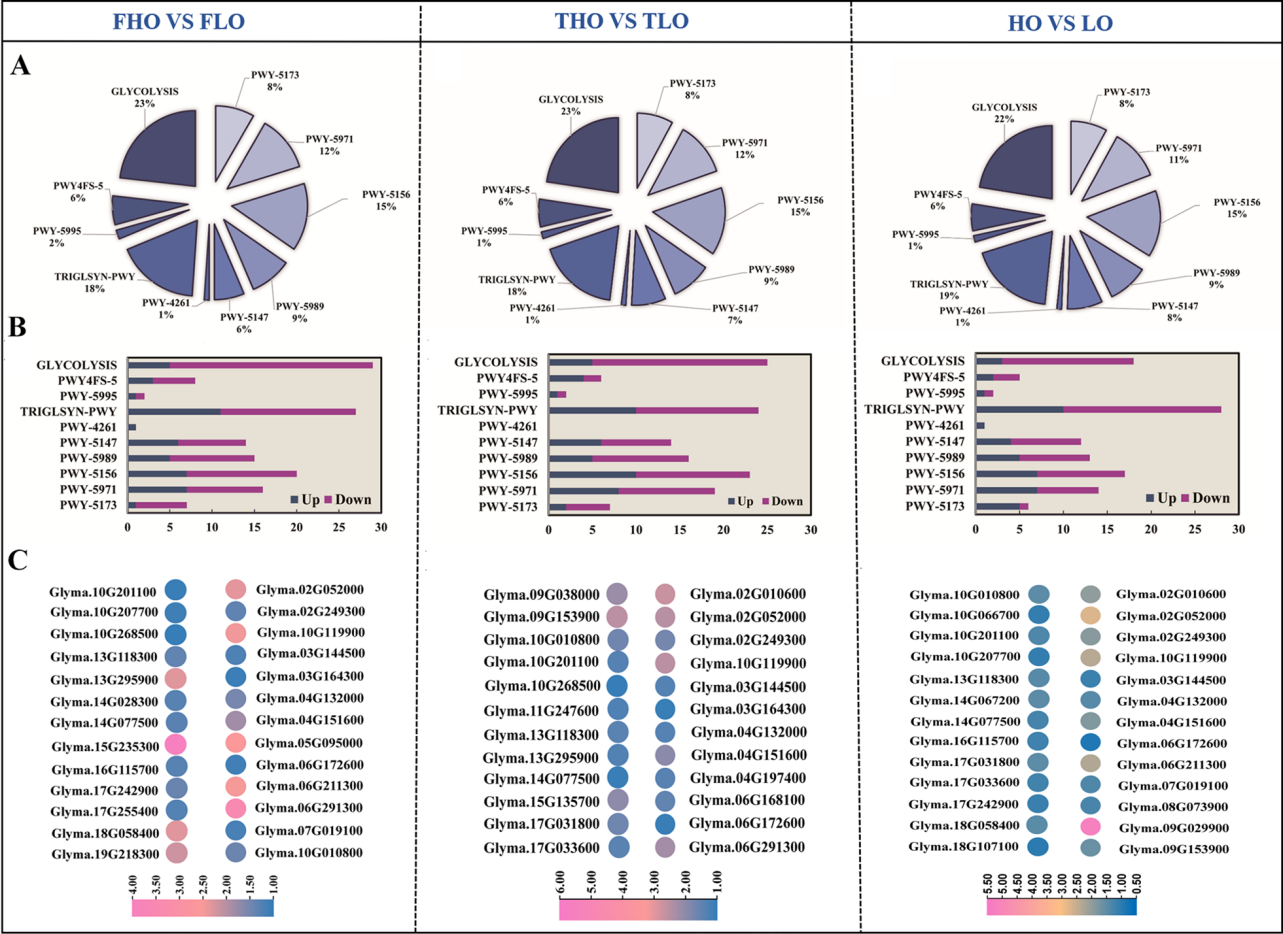
Functional categorization and expression analysis of lipid-related genes in the three comparison groups. The distribution of lipid-related DEGs identified in **A**. The functional categorization of lipid-related genes from **B** FHO vs. FLO, THO vs. TLO, and HO vs. LO. Differentially expressed genes (DEGs) involved in major lipid metabolism pathways in **C** FHO vs. FLO, THO vs. TLO, and HO vs*.* LO. GLYCOLYSI: glycolysis I (from glucose 6-phosphate), PWY4FS-5: superpathway of phosphatidylcholine biosynthesis, PWY-5995: linoleate biosynthesis I (plants), TRIGLSYN-PWY: diacylglycerol and triacylglycerol biosynthesis, PWY-4261: glycerol degradation I, PWY-5147: oleate biosynthesis I (plants), PWY-5989: stearate biosynthesis II (bacteria and plants), PWY-5156: superpathway of fatty acid biosynthesis II (plant), PWY-5971: palmitate biosynthesis II (bacteria and plants), and PWY-5173: superpathway of acetyl-CoA biosynthesis

Soybean fatty acid-related genes were identified from soybean (high- and low-oil content) transcriptome data according to the soybean genome database (https://soycyc.soybase.org/). The expression of fatty acid-related genes was assessed in each pathway. A total of 10 fatty acid-related pathways were detected, and the lipid-related genes were classified into specific pathways (Additional file 1: Table S2; Fig. 3A). Lipid-related genes with high expression were detected in “GLCOLYSIS,” “TRIGLSYN-PWY,” and “PWY-5156” in each comparison group (Fig. 3B). To evaluate the changes in lipid DEGs in each pathway among different comparison groups, a bar graph of lipid DEGs is shown in Fig. 3C. In different comparison groups, many lipid DEGs involved in “TRIGLSYN-PWY”, “PWY-5156”, and “PWY-5971” were identified as significantly upregulated, including diacylglycerol acyltransferase (Glyma.13G295900, DGAT), glycerol-3-phosphate 1-O-acyltransferase (Glyma.10G119900, GPAT), and very-long-chain acyl-CoA synthetase (Glyma.07G019100, LASC) (Fig. 3C). In addition, the glycolytic pathway may be involved in lipid metabolism, and GAPDH and PK were found to be highly expressed in each comparison group.

### Differential accumulation of lipids with HO and LO content

**Fig. 4:**
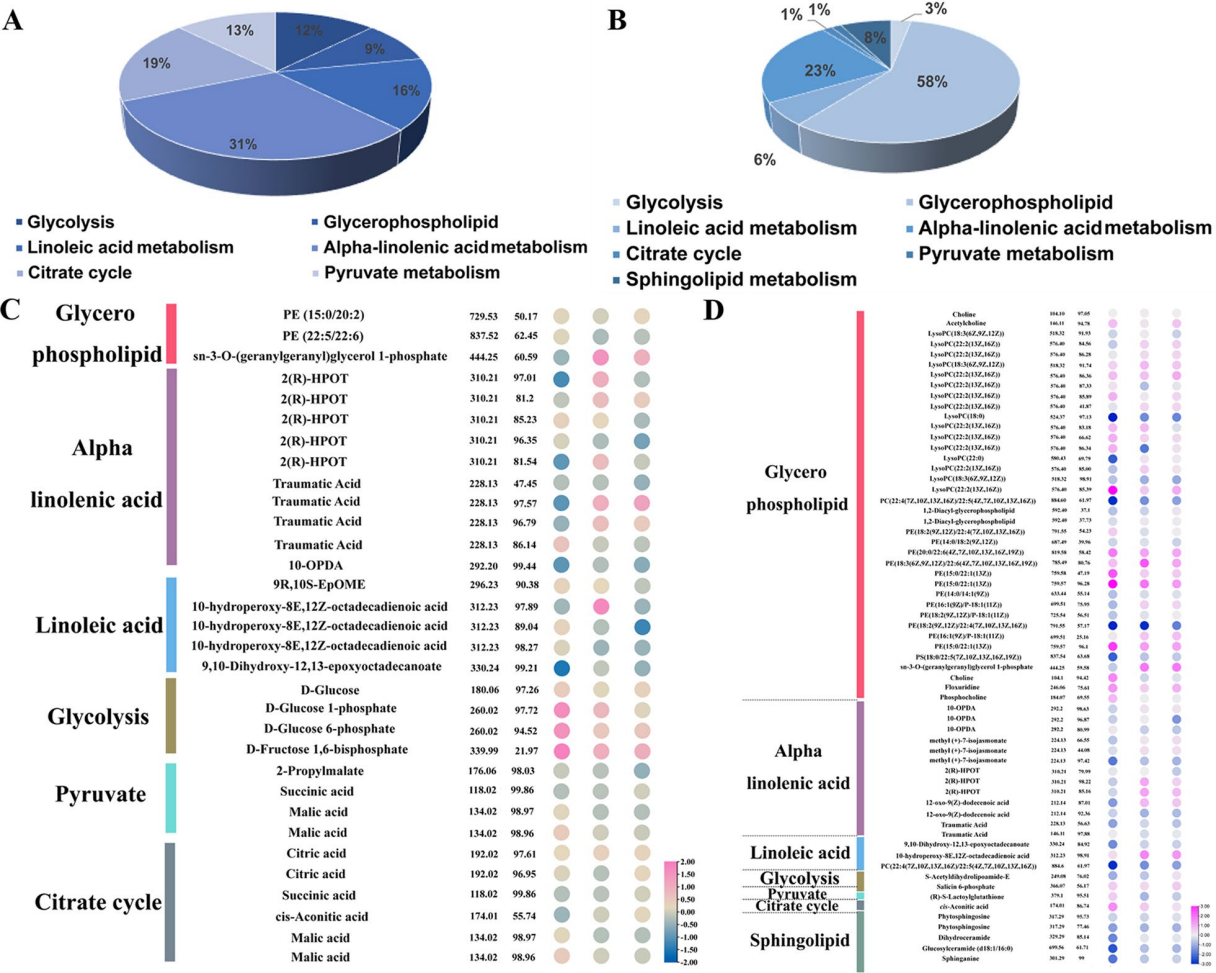
Differential metabolite analysis in the three comparison groups in positive ion mode and negative ion mode. **A** The distribution of lipid-related metabolites identified in negative ion mode. **B** The distribution of lipid-related metabolite identified in positive ion mode. **C** Enrichment analysis of lipid-related metabolites in the three comparison groups in negative ion mode. **D** Enrichment analysis of lipid-related metabolites in the three comparison groups in ion positive mode

The lipids in soybean play essential roles in regular cell functioning. In this study, the lipid-related metabolites of 30 soybean varieties during the respective R6 periods were studied. In the negative ion mode, a total of 32 lipid-related metabolites were discovered using the KEGG database, and these were classified into six metabolic pathways. Most of the alpha-linolenic acid-related metabolites were enriched in negative ion mode (Fig. 4A; Additional file 1: Table S3). In the glycolysis metabolic pathway, there were three DAMs in FMHO vs. FMLO, namely D-glucose 1-phosphate, D-glucose 6-phosphate, and D-fructose 1,6-bisphosphate. There was one DAM in the glycolysis metabolic pathway in TMHO vs. TMLO and MHO vs. MLO. In the linoleic acid and alpha-linolenic acid metabolic pathways, 10-hydroperoxy-8E,12Z-octadecadienoic acid, 9,10-dihydroxy-12,13-epoxyoctadecanoate, 2(R)-HPOT, and traumatic acid were found to be differentially accumulated in different comparison groups (Fig. 4C).

In the positive ion model, a total of 66 lipid-related metabolites were identified, and among these, the glycerophospholipid pathway had the most annotated metabolites (Fig. 4B). A cluster heatmap of 66 lipid-related metabolites showed that glycerophospholipid pathway metabolites accumulated significantly in the three comparison groups (FMHO vs. FMLO, TMHO vs. TMLO, and MHO vs. MLO). In the FMHO vs. FMLO comparison group, LysoPC (22:2 (13Z, 16Z)), PE (20:0/22:6 (4Z, 7Z, 10Z, 13Z, 16Z, 19Z)), and PE (15:0/22:1(13Z)) were found to be significantly accumulated. In the TMHO vs. TMLO comparison group, PE (15:0/22:1 (13Z)), PE (20:0/22:6 (4Z, 7Z, 10Z, 13Z, 16Z, 19Z)), and PE (18:3(6Z, 9Z, 12Z)/22:6(4Z, 7Z, 10Z, 13Z, 16Z, 19Z)) were significantly enriched. In the MHO vs. MLO comparison group, LysoPC(22:2(13Z, 16Z)), LysoPC(22:2(13Z, 16Z)), PE (20:0/22:6(4Z, 7Z, 10Z, 13Z, 16Z, 19Z)), PE(18:3(6Z, 9Z, 12Z)/22:6(4Z, 7Z, 10Z, 13Z, 16Z, 19Z)), and PE(15:0/22:1(13Z)) were highly accumulated (Fig. 4D). These results showed that these lipid metabolites might play an important role in oil synthesis in soybean.

### Other relevant DAMs

Flavonoids and carbon and amino acids were also identified in this study, and the results were consistent with the oil content determination. Our results suggest that the accumulation of carbon and sugars might play an important role in soybean oil content and yield.

The flavonoid DAMs in the three comparison groups were also compared. A total of 57 flavonoids were found in the three comparison groups. As shown in Additional file 1: Figure S2, ( − )-maackiain-3-O-glucosyl-6′′-O-malonate, ( +)-gallocatechin, 5-O-caffeoylshikimic acid, 8-C-glucosylnaringenin, and butin were more highly accumulated in FMHO compared to FMLO. In the TMHO vs. TMLO comparison group, 21 flavonoids were upregulated. However, no flavonoids were significantly enriched in the MHO vs. MLO comparison group (Additional file 1: Figure S2). The results suggest that the accumulation of flavonoids may affect the seed coat color and yield of soybean.

### Combined analysis of gene–metabolite network reveals the biosynthesis mechanism of lipids in HO and LO varieties

The KEGG enrichment analysis result showed that the DEMs and DEGs were enriched in photosynthesis, fatty acid biosynthesis, linoleic acid metabolism, and flavonoid biosynthesis pathways in the three comparison groups (Additional file 1: Figure S3).

**Fig. 5:**
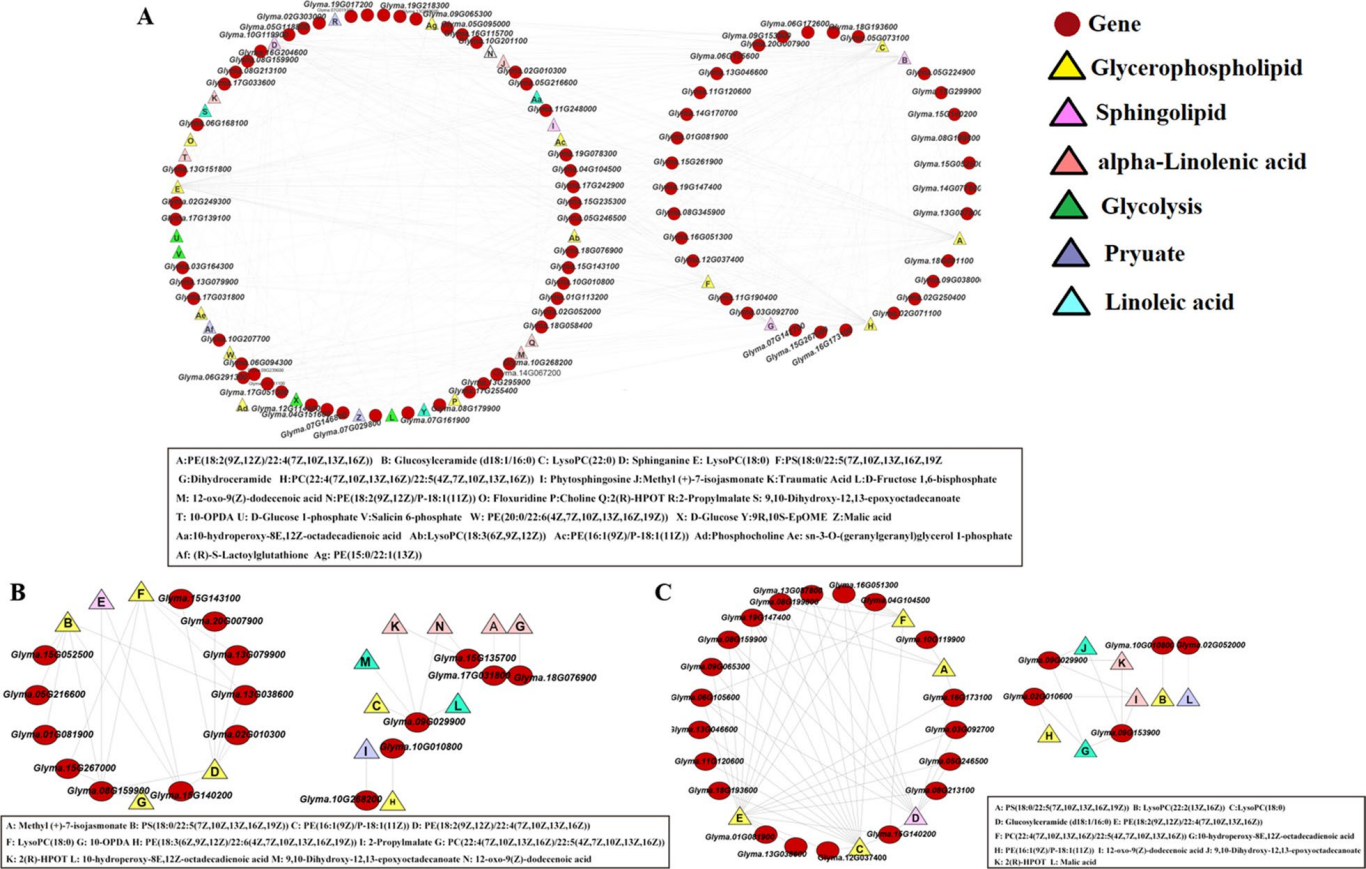
Network analysis of DEGs and DAMs in the three comparison groups. **A** FHO vs. FLO, **B** THO vs. TLO, and **C** HO vs. LO. Red circles represent different genes. Triangles with different colors represent different metabolic pathways

Gene and metabolite networks were constructed. In the FHO vs. FLO network, 33 lipid-related metabolites and 83 lipid-related genes generated 212 subnetworks (*r* > 0.5). The results showed that the 14 subnetworks were significantly correlated (*r* > 0.8, *p* < 0.01). LysoPC(18:0) was found to be positive associated with *Glyma.15G140200* (*r* > 0.91, *p* < 1.52E-08) and *Glyma.10G119900* (*r* > 0.94, *p* < 1.73E-10). Sphinganine was found to be negatively associated with *Glyma.17G033600* (*r* <   − 0.61, *p* < 0.004) *Glyma.06G172600* (r <  − 0.60, *p* < 0.004), and *Glyma.17G242900* (r <   − 0.59, *p* < 0.005) (Fig. 5A).

In the THO vs. TLO network, 14 lipid-related metabolites and 17 lipid-related genes generated 35 subnetworks (*r* > 0.5). Among them, important regulatory lipid-related genes and lipid-related metabolites were discovered, including *Glyma.09G029900*, which was positively associated with 12-oxo-9(Z)-dodecenoic acid (*r* > 0.87, *p* < 2.90E-13) and 10-hydroperoxy-8E,12Z-octadecadienoic acid (*r* > 0.89, *p* < 3.93E-15) (Fig. 5B).

In the HO vs. LO network, 12 lipid-related metabolites and 25 lipid-related genes generated 63 subnetworks (*r* > 0.5). In the subnetwork, LysoPC (18:0) and glucosylceramide (d18:1/16:0) metabolites were significantly associated with multiple lipid-related genes (Fig. 5C; Additional file 1: Table S4). This result showed that these metabolites might play important roles in oil synthesis.

### Co-expression analysis of transcription factors and lipid-related metabolites

In this study, transcription factors (TFs) were obtained from online databases (http://planttfdb.cbi.pku.edu.cn/), and differentially expressed TFs were identified using transcriptome data. In FHO vs. FLO, THO vs. TLO, and HO vs. LO, a total of 1110, 986, and 2165 TFs were screened, respectively. The most abundant TF families in each comparison group were bHLH, MYB, and ERF (Additional file 1: Figure S4).

**Fig. 6:**
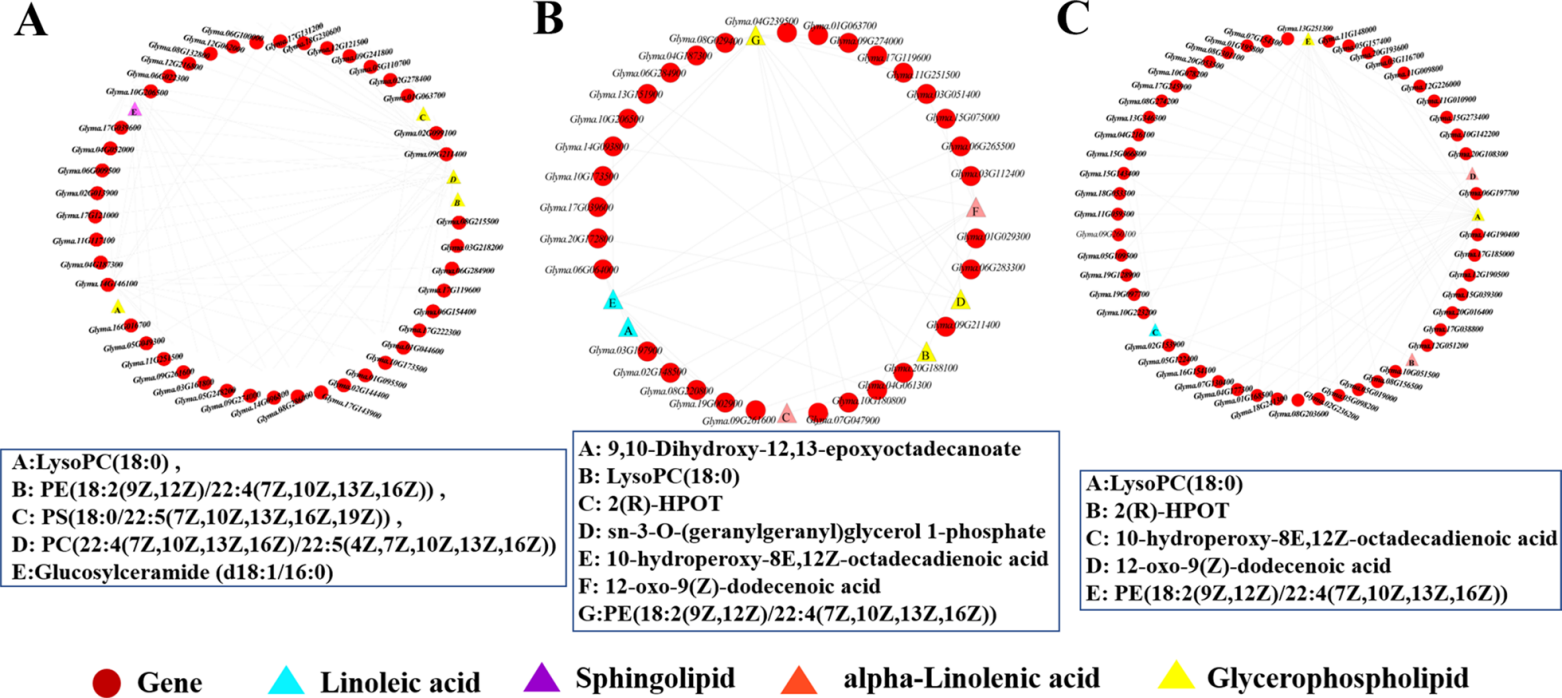
Network analysis of transcription factors and lipid metabolites in the three comparison groups. **A** FHO vs. FLO, **B** THO vs. TLO, and **C** HO vs. LO. Red circles represent different transcription factors. Triangles with different colors represent different metabolic pathways

In the FHO vs. FLO network, 42 TFs and five lipid-related metabolites generated 66 subnetworks (*r *> 0.92). Among them, the *GmMYB*s (eight) and *GmbHLHs* (eight) genes were found to be most abundant in the subnetworks (Fig. 6A; Additional file 1: Table S5). There were 31 TFs involved in regulating lipid metabolites in THO vs. TLO (*r* > 0.8), and 50 TFs were identified as being related to lipid metabolites in HO vs. LO (*r* > 0.7), which all contained different members of TFs, such as MYB (*Glyma.04G177300*, *Glyma.05G098200*, *Glyma.10G142200*, *Glyma.11G010900* and *Glyma.15G066800*), bHLH (*Glyma.08G203600*, *Glyma.08G274200*, *Glyma.13G251300* and *Glyma.19G128900*), and AP2/ERF (*Glyma.03G116700*, *Glyma.05G157400*, *Glyma.10G223200* and *Glyma.16G154100*). This result indicated that these TFs might play key roles during oil synthesis in soybean (Fig. 6).

### Combined analysis of the gene–metabolite network reveals the biosynthesis mechanism of lipids in HO and LO soybean seeds

**Fig. 7:**
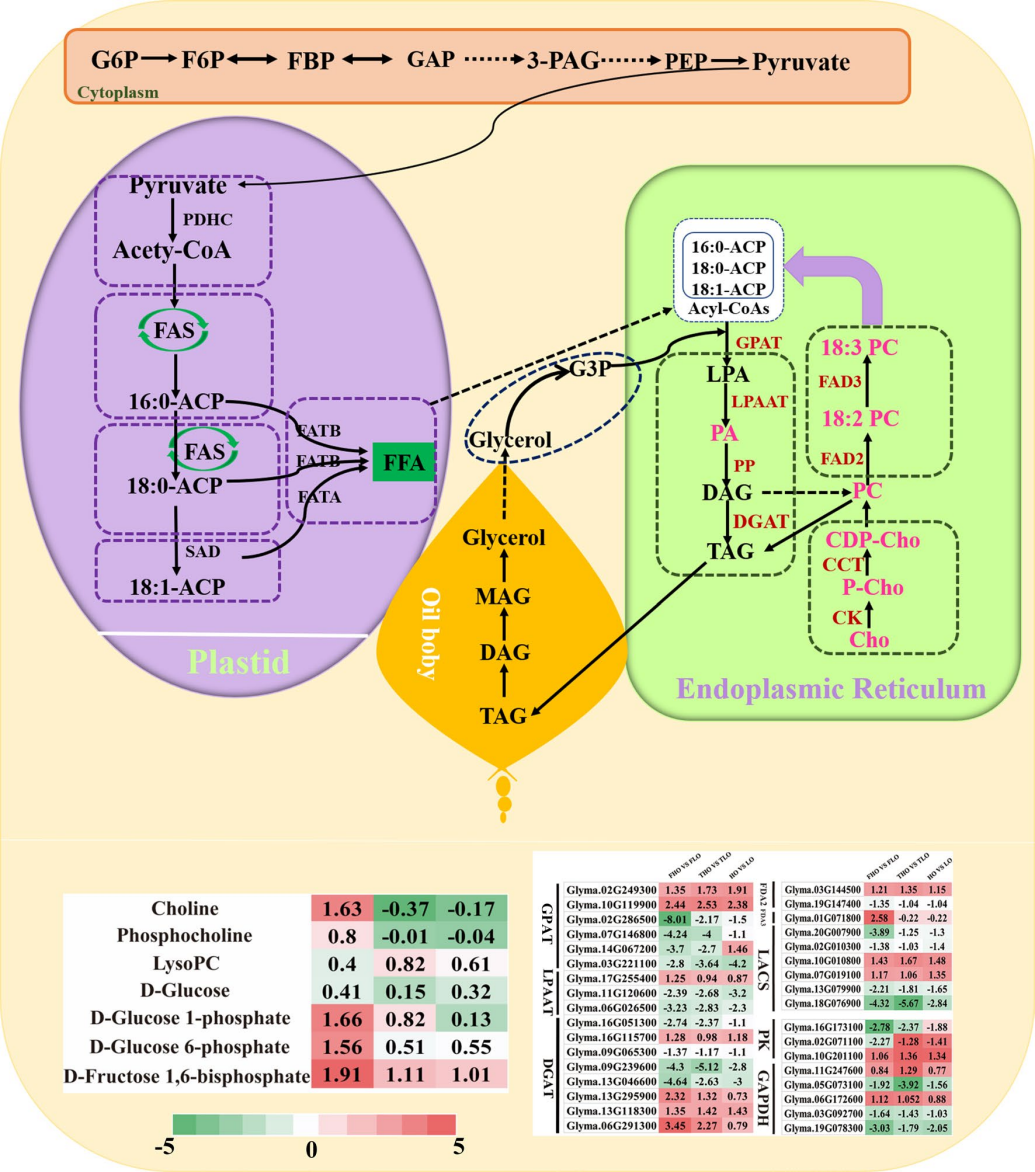
Gene–metabolite network illustrating lipid metabolism in low-oil and high-oil soybean. The oil synthesis pathways are displayed, and the involved genes and metabolites are indicated. Red represents higher expression levels, and green represents lower expression levels

The differences in lipid synthesis in the seeds of the three comparison groups were explored based on the integrated analysis of the transcriptomics and metabolomics data. As shown in Fig. 7, lipid biosynthesis pathways were analyzed in this study, which mainly included glycolysis, fatty acid synthesis, and the Kennedy pathway.

Glycolysis mainly provides a carbon supply for vegetable oil synthesis. In this study, the D-glucose content was reduced compared to D-glucose 6-phosphate and D-fructose 1,6-bisphosphate. The *GmFBP*, *GmGAPDH*, *GmPK*, and Gm*PFK* genes were found to be upregulated. These findings indicated the possible reason of the decrease in glucose content is that the glucose is being used as a substrate for lipid synthesis.

The de novo production of TAGs is through the Kennedy pathway and is catalyzed by LPAT, PAH, and DGAT. In the ER, the LPAT, DGAT, and PAH are the rate-limiting enzymes in TAG synthesis [25, 26]. In the three comparison groups, *GmLPAAT*, *GmGPAT*, and *GmDGAT* were markedly induced.

### Quantitative RT-PCR validation

Ten DEGs were randomly selected for qRT-PCR analysis to further verify the reliability of the RNA-seq results. The relative expression levels of the 10 genes in the qRT-PCR were consistent with the transcriptome results data (Additional file 1: Figure S5). The expression profiles of the 10 genes were obtained by qRT-PCR, and the transcriptome results indicated significant correlations in FHO vs. FLO (*R*^2^ = 0.79), THO vs. TLO (*R*^2^ = 0.76), and HO vs. LO (*R*^2^ = 0.79) (Additional file 1: Figure S6). The above results showed that the transcriptome data in this study were reliable.

## Discussion

Soybean oil is valued as an edible vegetable oil as well as for industrial applications and biofuels [27]. Previous studies have shown that plant oil is stored as TAGs [28]. In plants, glycolysis pathway provides carbon source for fatty acid synthesis and further generates TAG [29, 30]. In this study, a combined metabolomics and transcriptomic approach was used to explore the metabolite changes and the transcriptional regulation in HO and LO soybean varieties.

Previous studies have suggested that the most important lipid compounds in soybean are glycerophospholipids, primarily PC, PE, and PA [31]. Some of these metabolic compounds have been found in soybean [32]. In this study, we found that 98 lipid-related metabolites could be classified into six metabolic pathways (Additional file 1: Table S3). In FMHO vs. FMLO, there were three DEMs that were significantly enriched in the glycolysis metabolic pathway, namely D-glucose 1-phosphate, D-glucose 6-phosphate, and D-fructose 1,6-bisphosphate (Fig. 4). In the parallel transcriptomic analysis, glycolytic pathway genes were significantly upregulated, including the *GAPDH*, *PK*, and *BASS* genes. It is reported that *Bass2* can increase the oil content of *Brassica napus* [33]. Some researchers have revealed that GAPCs can regulate the accumulation of seed oil content [34]. In *Arabidopsis*, over-expression of *AtPKp* gene increased seed oil content [35]. Previous studies found that WRI1 is a major regulator in the glycolytic pathway and lipid metabolism [36–38]. Previous studies exhibited that glycolysis metabolites are closely related to seed oil content, such as fructose-6-phosphate (F6P), glucose-6-phosphate (G6P) and fructose-1,6-diphosphate (FBP), etc. [33]. In the TMHO vs. TMLO and MHO vs. MLO comparison groups, we also found that glycolysis metabolites were enriched. The above results showed that the glycolysis pathway provides a carbon source for oil synthesis.

The main component of soybean oil is TAGs, which are synthesized from G3P precursors [39]. In this study, we found that glycerophospholipids were the main components of lipid-related metabolites. A total of 38 lipid-related metabolites in the glycerophospholipid pathway were identified (Additional file 1: Table S3). In FMHO vs. FMLO, 11 lipid DAMs in the glycerophospholipid pathway were found. Among these, LysoPC (22:2(13Z, 16Z)) and PE (15:0/22:1(13Z)) were significantly enriched. In the parallel transcriptomic analysis, we found that *GmLAAPT* and *GmGPAT* were upregulated (Additional file 1: Table S2). Previous research has shown that DGAT and GPAT are involved in TAG biosynthesis [20, 21, 40]. In TMHO vs. TMLO and MHO vs*.* MLO, nine and 11 DAMs of lipids in the glycerophospholipid pathway were found, respectively. Among these, PE (15:0/22:1(13Z)) and LysoPC (22:2(13Z, 16Z)) were also significantly enriched. Previous studies exhibited that phosphatidylcholine (PC) is the most abundant phospholipid and plays a key role in the production of TAG [41, 42]. We deduce that the glycerophospholipid pathway may regulate the synthesis of TAGs.

To explore the relationship between genes and metabolites, a two-dimensional network diagram was constructed using lipid-related genes and metabolites. A total of 212 subnetworks were identified in the FMHO vs. FMLO comparison group. Multiple studies have demonstrated that genes and metabolites related to the glycolysis pathway affect the accumulation of plant oil [33, 35]. Glycolysis is core to the synthesis of oil, as it converts sugars into precursors for the synthesis of fatty acids [35]. In this work, LysoPC(18:0) was found to be positively associated with *Glyma.10G119900* (*GmGPAT*) (*r* > 0.94, *p* < 1.73E-10). It is reported that LysoPC is the main component of fatty acid synthesis, and *GmGPAT* is related to TAG synthesis [43, 44]. We also found that sphinganine was negatively associated with Glyma.06G172600 (*GmGAPDH*) (r <   −  0.60, *p* < 0.004). Sphinganine was downregulated, and the *GmGAPDH* gene was upregulated. Thus, *GmGPAT,* and *GmGAPDH* may be key genes in glycolysis and oil synthesis, which may help elucidate the genetic relationship between glycolysis and seed oil synthesis.

In conclusion, this combined metabolome and transcriptome study allowed for a large-scale analysis of lipids in soybean.

## Conclusions

A total of 5970 metabolites were identified using a non-targeted approach. We identified 98 lipid-related metabolites, including glycerophospholipids, alpha-linolenic acid, linoleic acid, glycolysis, pyruvate, and the sphingolipid pathway, which significantly broadens our understanding of the lipid compounds present in soybean. We further explored the correlation network and identified novel candidates (*GPAT* and* GAPDH*) that regulate lipid biosynthesis in soybean. The above results expand our understanding of lipid accumulation patterns and molecular regulatory mechanisms in soybean.

## Methods

### Plant materials

Thirty soybean varieties, comprising 15 with high-oil and 15 with low-oil contents, were evaluated in this study and were obtained from the Soybean Research Institute, Northeast Agricultural University. These 30 soybean varieties were grown under the same field conditions in Harbin (162.41°E, 45.45°N), Heilongjiang, China. The samples were collected at the R6 developmental stage, and two biological replicates were collected. All samples were frozen quickly in liquid nitrogen for transcription and metabolite analysis. These mature seeds were used to determine the oil content.

### Metabolite profiling

Non-targeted metabolome analysis was performed by Bioacme Biotechnology Co., Ltd. (Wuhan, China). Briefly, 100 mg of sample was placed into a 1.5-mL centrifuge tube, to which 300 μL of 75% methanol/water was added and centrifuged at 12,000 rpm, 10 min at 4 °C. All metabolites were identified using the Metlin database. The differential metabolites were analyzed using an orthogonal partial least squares-discriminant analysis (OPLS-DA) model, with a variable importance in the projection (VIP) score of ≥ 1 and a |log2 (fold change)| of ≥ 1. The functional annotations of these metabolites were obtained using the Kyoto Encyclopedia of Genes and Genomes (KEGG) database (http://www.kegg.jp/kegg/compound/).

### Transcriptome sequencing

Total RNA was isolated and an RNA library was constructed for each sample using an Illumina HiSeq platform by Bioacme Biotechnology Co., Ltd. (Wuhan, China). Raw sequences were obtained by removing the adapter sequence, low-quality reads, and poly-N. Clean data quality is controlled using FastQc (V0.11.8) software [45]. Q20, Q30 and GC-content of the clean data were calculated. The adaptor and low-quality sequence reads were deleted from the data sets. After data processing, the Raw sequences were converted into clean reads. The high-quality clean reads were mapped to the reference genome and were used for transcriptome analysis. Hisat2 software were applied to map with reference genome [46]. The unigenes were annotated by searching the Swiss-Prot, Gene Ontology (GO), Eukaryotic Orthologous Groups of proteins (KOG), Non-redundant (NR), and KEGG databases. Differentially expressed genes (DEGs) were identified using the edgeR R package [47]. A |log2 (fold change)| of ≥ 1 and a false discovery rate of < 0.05 were used to define significant differential expression. 10 fatty acid-related pathways were identified from the soybean genome database (https://www.soybase.org/).

### Gene–metabolite network analysis

The transcription factor was screened using the online database (http://planttfdb.cbi.pku.edu.cn/). Differential transcription factors and lipid-related genes are identified (|log2 (fold change)| of ≥ 1). Transcription factors, lipid-related genes and lipid-related metabolites were used to construct network relationship in R, respectively. And a Pearson’s correlation cutoff value of 0.5 was generated. Visualization of the network was performed using Cytoscape 3.6.0 software [48].

### Quantitative real-time PCR

Several DEGs were subjected to quantitative real-time PCR (qRT-PCR) analysis. The RNA was extracted and cDNAs were generated with ReverTra Ace qPCR RT Master Mix (TOYOBO, Osaka, Japan). The qRT-PCR was accomplished by CFX Connect TM real-time system (BIO-RAD) with the SYBR Green PCR kit (SYBR Green, TOYOBO, Osaka, Japan). *GmACTIN* was used as an internal control. The DN50 seed samples were used as a calibrator. Three biological replicates with three technical replicates were applied to each sample. Relative expression levels were estimated using the 2^−ΔΔct^ method [49]. All qRT-PCR primers are listed in Additional file 1: Table S6.

### Statistical analysis

All data were analyzed using Excel 2019 (Microsoft Corp., Redmond, WA, USA) and SPSS 19.0 (IBM Corp., Armonk, NY, USA), and significance tests were achieved by Student’s* t*-test.

## Supplementary Information

**Additional file 1: Figure S1.** The oil content of the 30 soybean varieties. Student’s *t*-test were carried the significance levels (*P<0.05, **P<0.01). **Figure S2.** The content of flavonoid-related metabolites in the three comparison groups. Red represents up-regulated, and blue represents down-regulated. **Figure S3.** KEGG enrichment analysis *p-*value histogram of the differentially expressed genes (DEGs) and differentially abundant metabolites (DAMs) of the three comparison groups. **A**. FHO vs. FLO, **B**. THO vs. TLO, **C**. HO vs. LO. Blue represents gene, and green represents metabolite. **Figure S4.** **A**. Total number of DEG TFs in the three comparison groups. **B**. Number of various DEG TFs in the three comparison groups. **Figure S5.** Expression levels of 10 candidate genes in DN47 and DN50 soybean germplasms at the R6 growth period. Purple column represents DN50, blue column represents DN47. Student’s *t*-test were carried the significance levels (*P<0.05, **P<0.01). **Figure S6.** Correlations of the expression levels of the qRT-PCR and transcriptome data in the three comparison groups. **A**. FHO vs. FLO, **B**. THO vs. TLO, **C**. HO vs. LO. **Table S1.** Oil content of the 30 soybean varieties. **Table S2.** Statistics of differential genes related to oil synthesis. **Table S3.** Classification of metabolites related to lipid synthesis. **Table S4.** Co-expression analysis of lipid-related metabolites and genes. **Table S5.** Co-expression analysis of transcription factor and lipid-related metabolites. **Table S6.** Primers used for qRT-PCR.

## Data Availability

All data generated or analyzed during this study are included in this published article and its additional files.

## Abbreviations

FHO vs FLO: Five high-oil varieties vs five low-oil varieties
THO vs TLO: 10 High-oil varieties vs 10 low-oil varieties
HO vs LO: 15 High-oil varieties vs 15 low-oil varieties
ER: Endoplasmic reticulum
TAG: Triacylglycerols
G3P: Glycerol-3-phosphate
GPAT: Glycerol phosphate acyltransferase
LPAAT: Lysophosphatidic acid acyltransferase
DGAT: Diacylglycerol acyltransferase
PDAT: Phospholipid acyltransferase
LACS: Long-chain acyl-CoA synthetase
GAPDH: Glyceraldehyde-3-phosphate dehydrogenase
PK: Pyruvate kinase
LysoPC: Lysophosphatidylcholine
PE: Phosphatidylethanolamine
PAH: PA phosphatase
PA: Phosphatidic acid
FBP: Fructose-1,6-bisphosphatase
PFK: 6-Phosphofructokinase
