# Supplementary figures and images for: Combined analysis of the metabolome and transcriptome provides insight into seed oil accumulation in soybean
Source: Biotechnol Biofuels Bioprod. 2023 Apr 25;16:70. doi: 10.1186/s13068-023-02321-3 (PMC10131312; doi:10.1186/s13068-023-02321-3)

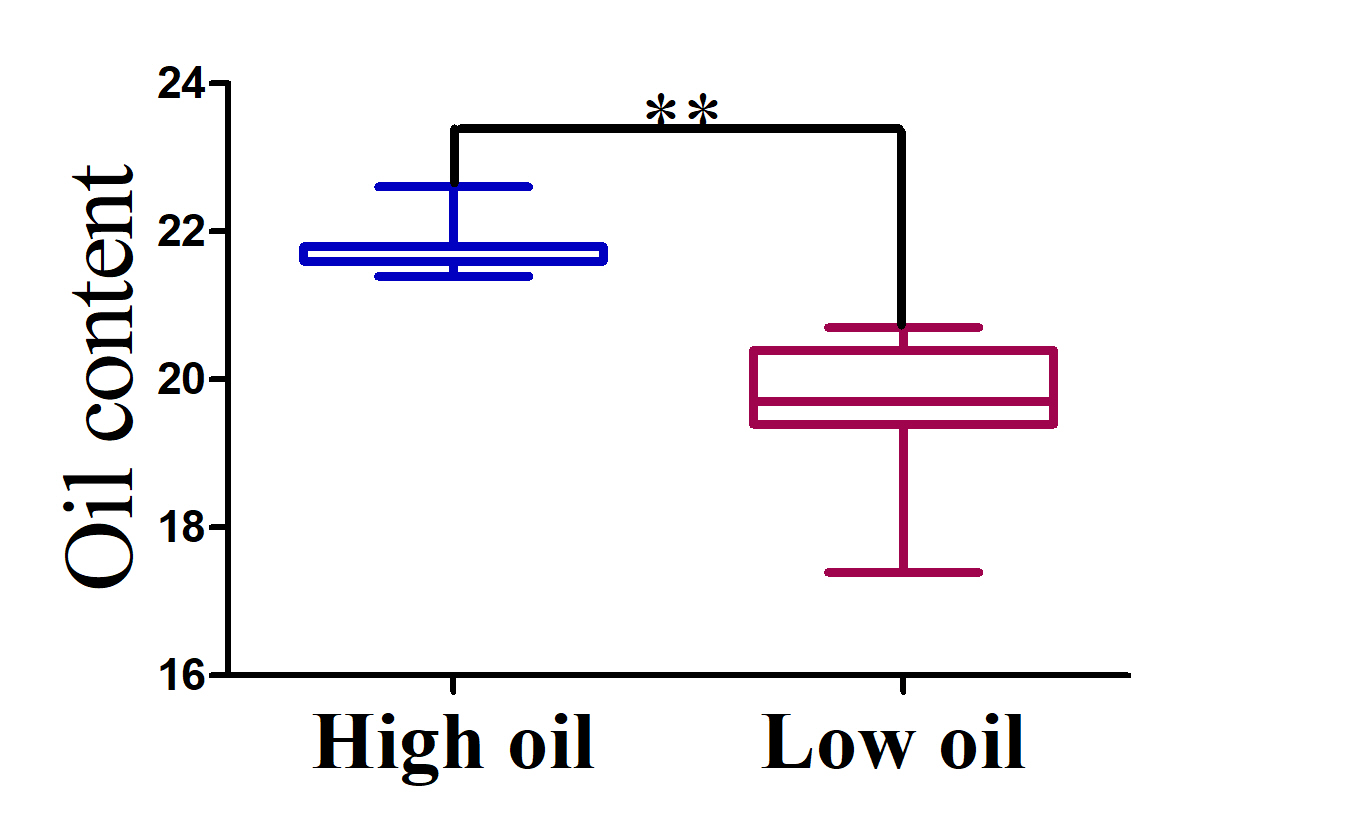

Supplement: Supplementary file 1 — Additional file 1: Figure S1. The oil content of the 30 soybean varieties. Student’s t-test were carried the significance levels (*P<0.05, **P<0.01). Figure S2. The content of flavonoid-related metabolites in the three comparison groups. Red represents up-regulated, and blue represents down-regulated. Figure S3. KEGG enrichment analysis p-value histogram of the differentially expressed genes (DEGs) and differentially abundant metabolites (DAMs) of the three comparison groups. A. FHO vs. FLO, B. THO vs. TLO, C. HO vs. LO. Blue represents gene, and green represents metabolite. Figure S4. A. Total number of DEG TFs in the three comparison groups. B. Number of various DEG TFs in the three comparison groups. Figure S5. Expression levels of 10 candidate genes in DN47 and DN50 soybean germplasms at the R6 growth period. Purple column represents DN50, blue column represents DN47. Student’s t-test were carried the significance levels (*P<0.05, **P<0.01). Figure S6. Correlations of the expression levels of the qRT-PCR and transcriptome data in the three comparison groups. A. FHO vs. FLO, B. THO vs. TLO, C. HO vs. LO. Table S1. Oil content of the 30 soybean varieties. Table S2. Statistics of differential genes related to oil synthesis. Table S3. Classification of metabolites related to lipid synthesis. Table S4. Co-expression analysis of lipid-related metabolites and genes. Table S5. Co-expression analysis of transcription factor and lipid-related metabolites. Table S6. Primers used for qRT-PCR. [file 13068_2023_2321_MOESM1_ESM.zip › Supplementary material/FigureS1.jpg]

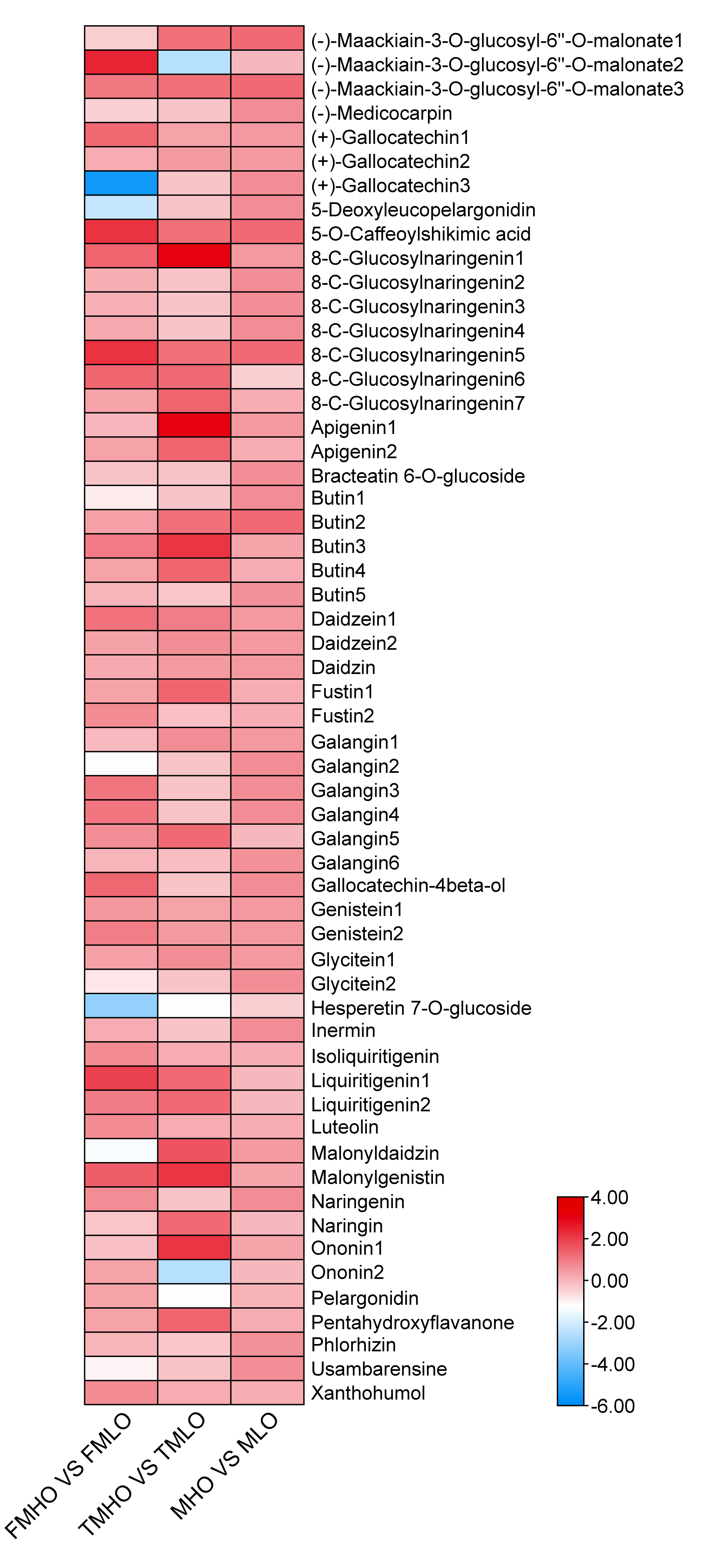

Supplement: Supplementary file 1 — Additional file 1: Figure S1. The oil content of the 30 soybean varieties. Student’s t-test were carried the significance levels (*P<0.05, **P<0.01). Figure S2. The content of flavonoid-related metabolites in the three comparison groups. Red represents up-regulated, and blue represents down-regulated. Figure S3. KEGG enrichment analysis p-value histogram of the differentially expressed genes (DEGs) and differentially abundant metabolites (DAMs) of the three comparison groups. A. FHO vs. FLO, B. THO vs. TLO, C. HO vs. LO. Blue represents gene, and green represents metabolite. Figure S4. A. Total number of DEG TFs in the three comparison groups. B. Number of various DEG TFs in the three comparison groups. Figure S5. Expression levels of 10 candidate genes in DN47 and DN50 soybean germplasms at the R6 growth period. Purple column represents DN50, blue column represents DN47. Student’s t-test were carried the significance levels (*P<0.05, **P<0.01). Figure S6. Correlations of the expression levels of the qRT-PCR and transcriptome data in the three comparison groups. A. FHO vs. FLO, B. THO vs. TLO, C. HO vs. LO. Table S1. Oil content of the 30 soybean varieties. Table S2. Statistics of differential genes related to oil synthesis. Table S3. Classification of metabolites related to lipid synthesis. Table S4. Co-expression analysis of lipid-related metabolites and genes. Table S5. Co-expression analysis of transcription factor and lipid-related metabolites. Table S6. Primers used for qRT-PCR. [file 13068_2023_2321_MOESM1_ESM.zip › Supplementary material/FigureS2.tif]

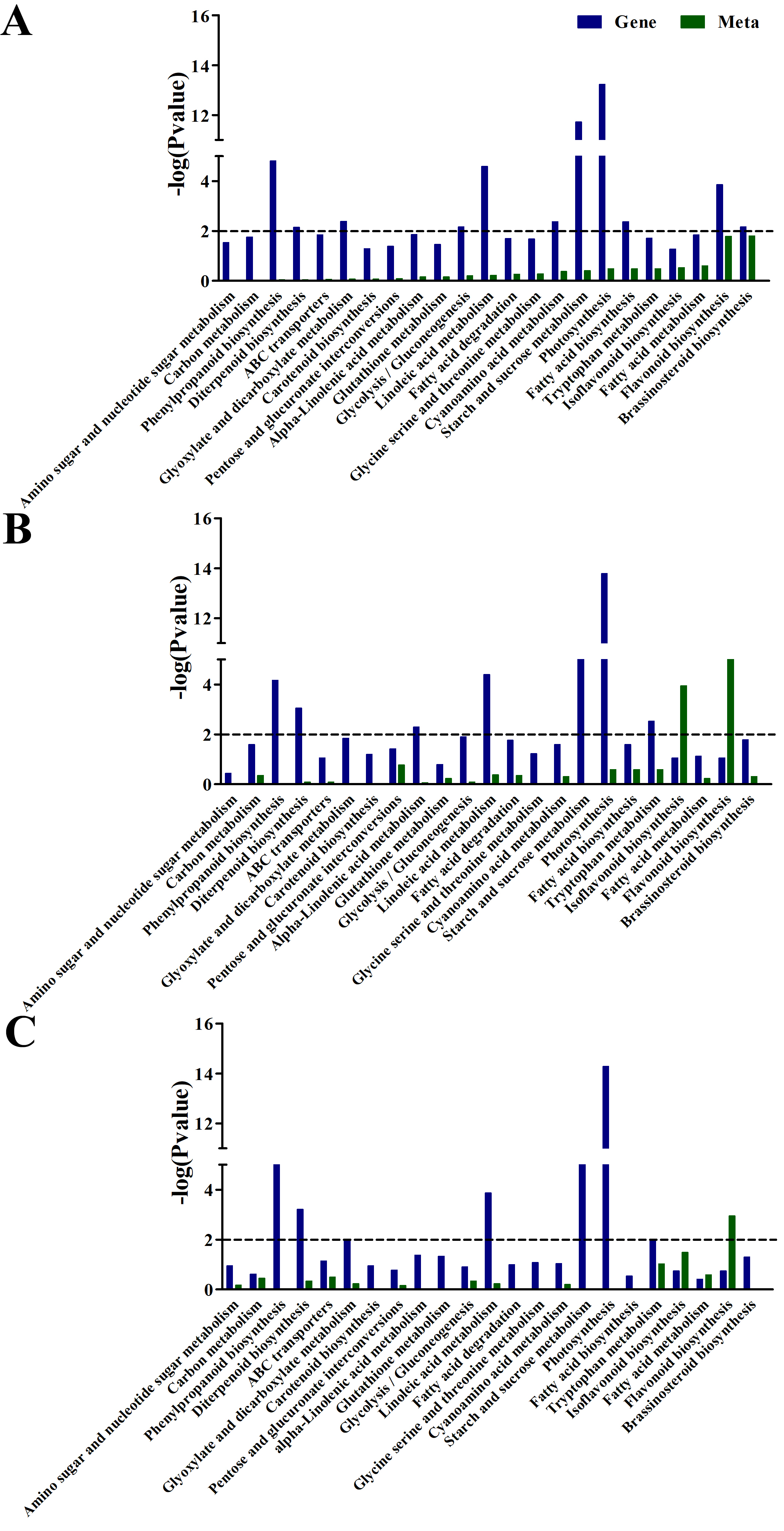

Supplement: Supplementary file 1 — Additional file 1: Figure S1. The oil content of the 30 soybean varieties. Student’s t-test were carried the significance levels (*P<0.05, **P<0.01). Figure S2. The content of flavonoid-related metabolites in the three comparison groups. Red represents up-regulated, and blue represents down-regulated. Figure S3. KEGG enrichment analysis p-value histogram of the differentially expressed genes (DEGs) and differentially abundant metabolites (DAMs) of the three comparison groups. A. FHO vs. FLO, B. THO vs. TLO, C. HO vs. LO. Blue represents gene, and green represents metabolite. Figure S4. A. Total number of DEG TFs in the three comparison groups. B. Number of various DEG TFs in the three comparison groups. Figure S5. Expression levels of 10 candidate genes in DN47 and DN50 soybean germplasms at the R6 growth period. Purple column represents DN50, blue column represents DN47. Student’s t-test were carried the significance levels (*P<0.05, **P<0.01). Figure S6. Correlations of the expression levels of the qRT-PCR and transcriptome data in the three comparison groups. A. FHO vs. FLO, B. THO vs. TLO, C. HO vs. LO. Table S1. Oil content of the 30 soybean varieties. Table S2. Statistics of differential genes related to oil synthesis. Table S3. Classification of metabolites related to lipid synthesis. Table S4. Co-expression analysis of lipid-related metabolites and genes. Table S5. Co-expression analysis of transcription factor and lipid-related metabolites. Table S6. Primers used for qRT-PCR. [file 13068_2023_2321_MOESM1_ESM.zip › Supplementary material/FigureS3.tif]

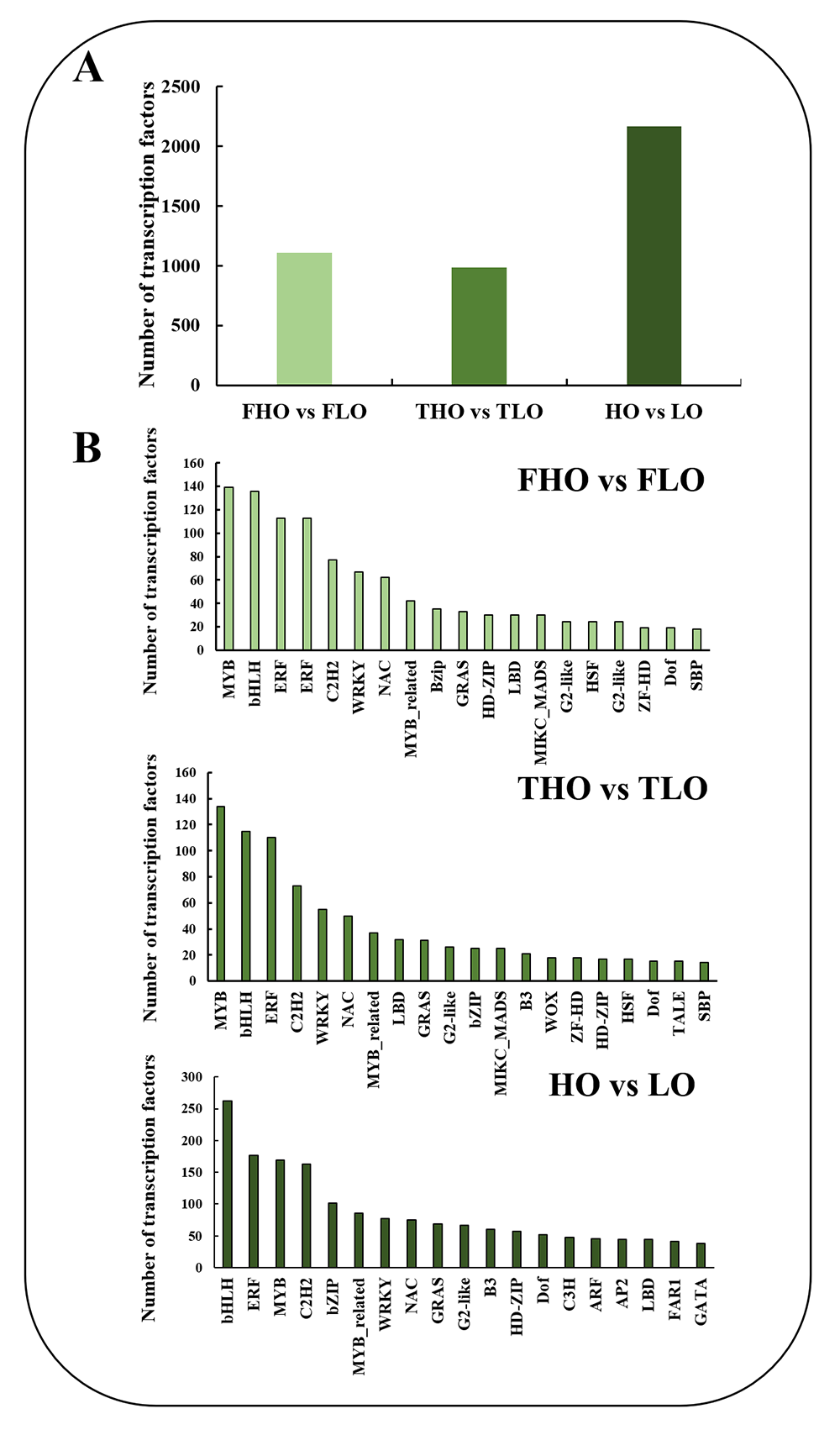

Supplement: Supplementary file 1 — Additional file 1: Figure S1. The oil content of the 30 soybean varieties. Student’s t-test were carried the significance levels (*P<0.05, **P<0.01). Figure S2. The content of flavonoid-related metabolites in the three comparison groups. Red represents up-regulated, and blue represents down-regulated. Figure S3. KEGG enrichment analysis p-value histogram of the differentially expressed genes (DEGs) and differentially abundant metabolites (DAMs) of the three comparison groups. A. FHO vs. FLO, B. THO vs. TLO, C. HO vs. LO. Blue represents gene, and green represents metabolite. Figure S4. A. Total number of DEG TFs in the three comparison groups. B. Number of various DEG TFs in the three comparison groups. Figure S5. Expression levels of 10 candidate genes in DN47 and DN50 soybean germplasms at the R6 growth period. Purple column represents DN50, blue column represents DN47. Student’s t-test were carried the significance levels (*P<0.05, **P<0.01). Figure S6. Correlations of the expression levels of the qRT-PCR and transcriptome data in the three comparison groups. A. FHO vs. FLO, B. THO vs. TLO, C. HO vs. LO. Table S1. Oil content of the 30 soybean varieties. Table S2. Statistics of differential genes related to oil synthesis. Table S3. Classification of metabolites related to lipid synthesis. Table S4. Co-expression analysis of lipid-related metabolites and genes. Table S5. Co-expression analysis of transcription factor and lipid-related metabolites. Table S6. Primers used for qRT-PCR. [file 13068_2023_2321_MOESM1_ESM.zip › Supplementary material/FigureS4.tif]

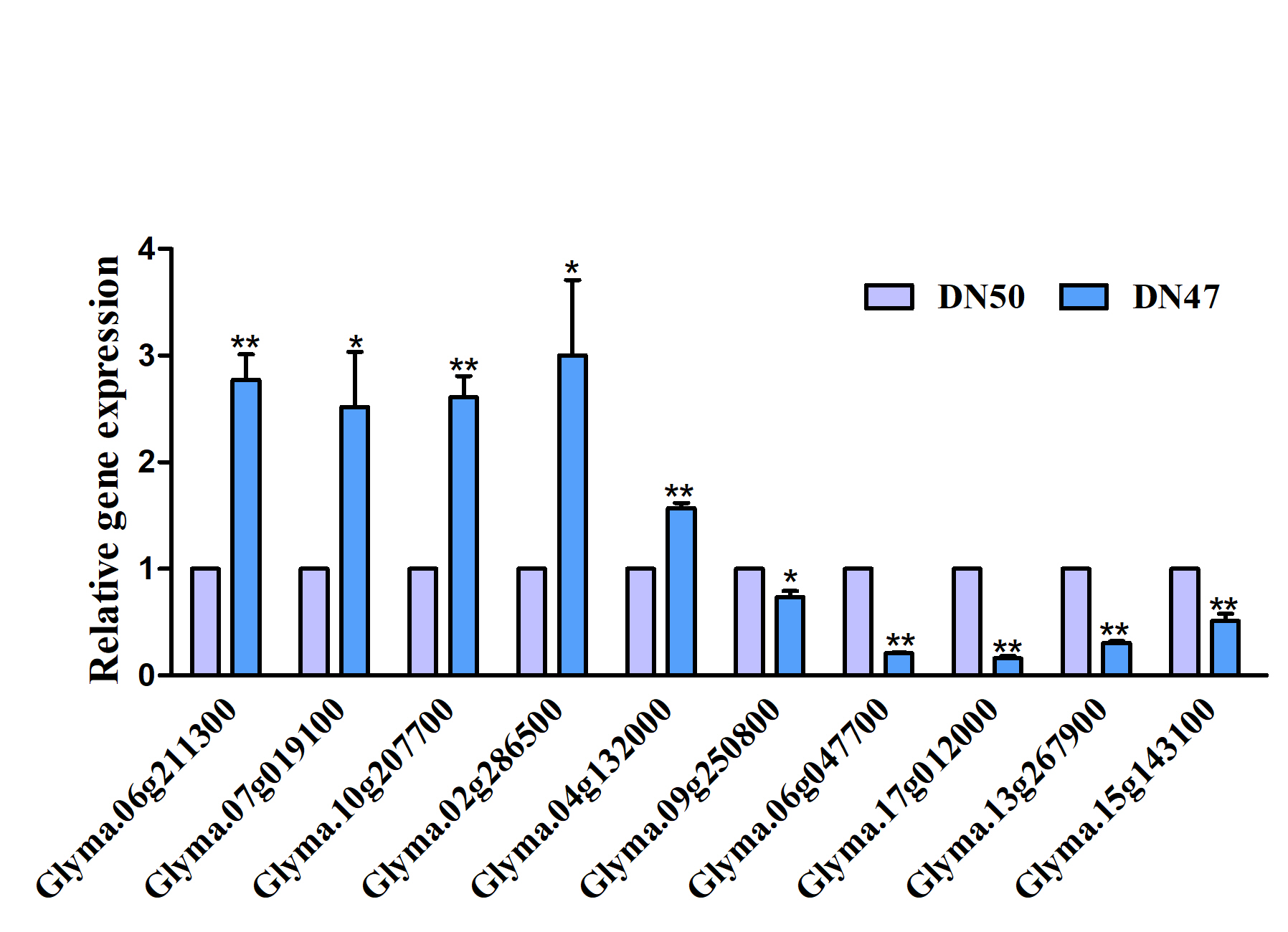

Supplement: Supplementary file 1 — Additional file 1: Figure S1. The oil content of the 30 soybean varieties. Student’s t-test were carried the significance levels (*P<0.05, **P<0.01). Figure S2. The content of flavonoid-related metabolites in the three comparison groups. Red represents up-regulated, and blue represents down-regulated. Figure S3. KEGG enrichment analysis p-value histogram of the differentially expressed genes (DEGs) and differentially abundant metabolites (DAMs) of the three comparison groups. A. FHO vs. FLO, B. THO vs. TLO, C. HO vs. LO. Blue represents gene, and green represents metabolite. Figure S4. A. Total number of DEG TFs in the three comparison groups. B. Number of various DEG TFs in the three comparison groups. Figure S5. Expression levels of 10 candidate genes in DN47 and DN50 soybean germplasms at the R6 growth period. Purple column represents DN50, blue column represents DN47. Student’s t-test were carried the significance levels (*P<0.05, **P<0.01). Figure S6. Correlations of the expression levels of the qRT-PCR and transcriptome data in the three comparison groups. A. FHO vs. FLO, B. THO vs. TLO, C. HO vs. LO. Table S1. Oil content of the 30 soybean varieties. Table S2. Statistics of differential genes related to oil synthesis. Table S3. Classification of metabolites related to lipid synthesis. Table S4. Co-expression analysis of lipid-related metabolites and genes. Table S5. Co-expression analysis of transcription factor and lipid-related metabolites. Table S6. Primers used for qRT-PCR. [file 13068_2023_2321_MOESM1_ESM.zip › Supplementary material/FigureS5.jpg]

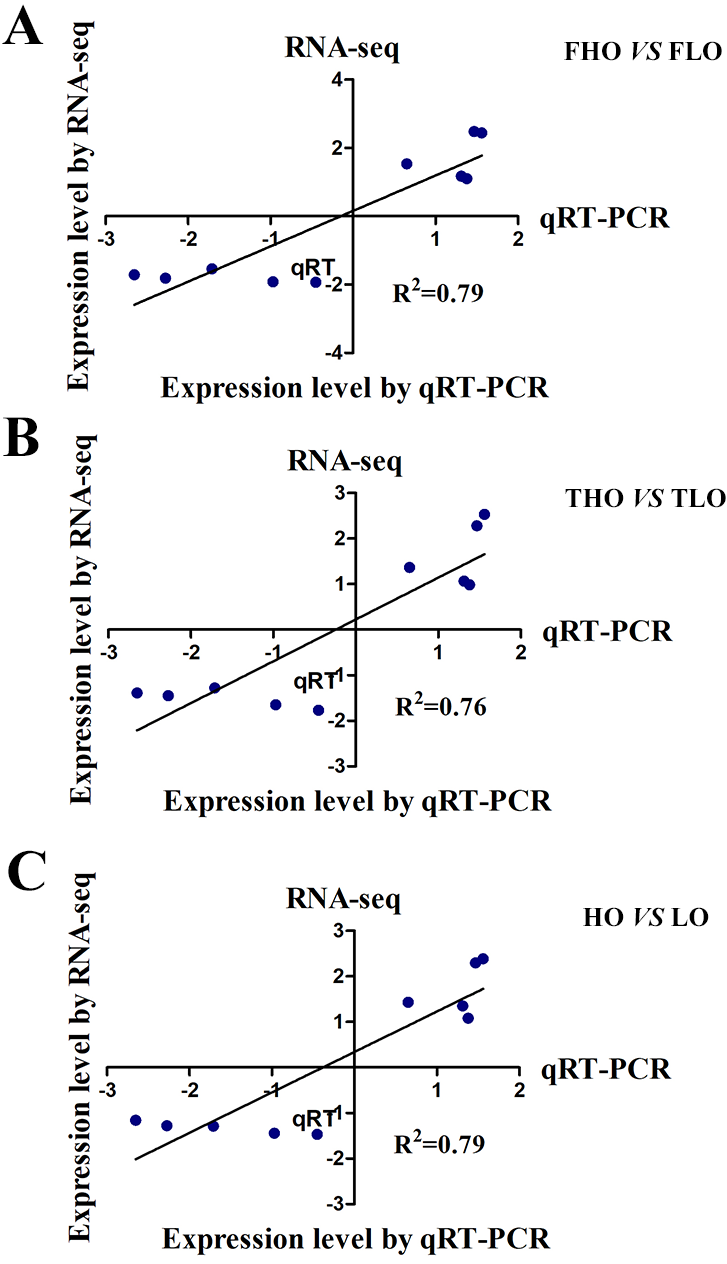

Supplement: Supplementary file 1 — Additional file 1: Figure S1. The oil content of the 30 soybean varieties. Student’s t-test were carried the significance levels (*P<0.05, **P<0.01). Figure S2. The content of flavonoid-related metabolites in the three comparison groups. Red represents up-regulated, and blue represents down-regulated. Figure S3. KEGG enrichment analysis p-value histogram of the differentially expressed genes (DEGs) and differentially abundant metabolites (DAMs) of the three comparison groups. A. FHO vs. FLO, B. THO vs. TLO, C. HO vs. LO. Blue represents gene, and green represents metabolite. Figure S4. A. Total number of DEG TFs in the three comparison groups. B. Number of various DEG TFs in the three comparison groups. Figure S5. Expression levels of 10 candidate genes in DN47 and DN50 soybean germplasms at the R6 growth period. Purple column represents DN50, blue column represents DN47. Student’s t-test were carried the significance levels (*P<0.05, **P<0.01). Figure S6. Correlations of the expression levels of the qRT-PCR and transcriptome data in the three comparison groups. A. FHO vs. FLO, B. THO vs. TLO, C. HO vs. LO. Table S1. Oil content of the 30 soybean varieties. Table S2. Statistics of differential genes related to oil synthesis. Table S3. Classification of metabolites related to lipid synthesis. Table S4. Co-expression analysis of lipid-related metabolites and genes. Table S5. Co-expression analysis of transcription factor and lipid-related metabolites. Table S6. Primers used for qRT-PCR. [file 13068_2023_2321_MOESM1_ESM.zip › Supplementary material/FigureS6.tif]
